# Supplementary material for: Structural insights into RNA bridging between HIV-1 Vif and antiviral factor APOBEC3G
Source: Nat Commun. 2023 Jul 7;14:4037. doi: 10.1038/s41467-023-39796-5 (PMC10328928; doi:10.1038/s41467-023-39796-5)
Supplement: Supplementary file 1 — Supplementary Information [file 41467_2023_39796_MOESM1_ESM.pdf]

Supplementary Information

Supplementary Note 1:

Preparation of a solubility-enhanced variant of human A3G

Kouno *et al*. prepared solubilized human A3G NTD (sNTD) and determined its solution structure using NMR spectroscopy [1]. Their structure-guided experiments identified amino acid determinants for sNTD-Vif interaction, which are widely distributed over the sNTD surface. We have expanded our structural analysis using the sNTD variant in a complex with Vif-CBFβ-ELOB-ELOC (VCBC). Co-expression experiments using variants of sNTD and VCBC demonstrated that the wild-type amino acid F126 of A3G enhances complex formation [1]. Indeed, the full-length construct, sNTD-F126-CTD, suffers Vif-induced degradation, as does wild-type A3G [1]. Although sNTD-F126 forms a stable complex with VCBC, sNTD-F126 prevented structure analysis due to its poor solubility and purification profile (Supplementary Fig. 1c), which suggested the necessity for a solubility-enhanced A3G variant.

To improve the purification profile, we focused on the sNTD β1/β2 loop region (Supplementary Fig. 1a, b). The solution structure showed that sNTD contains a relatively long loop between β1 and β2, compared with those of other A3s [1]. HSQC signals of amino acids in the loop of sNTD, L49, D50, and A51, were clearly split, implying that this loop structure underwent slow perturbation on a timescale of micro- to milli-seconds. To stabilize the conformation, amino acids of the β1/β2 loop were replaced with those of the "consensus" construct (Supplementary Fig. 1a) [1]. The newly prepared full-length A3G variant, sA3G, and its NTD alone could be fractionated into a single peak by size-exclusion chromatography (Fig. 1c and Supplementary Fig. 1d). Importantly, sA3G is sensitive to Vif-induced degradation, which can be attenuated using the proteasome inhibitor, MG132, or by the sA3G mutation D128K (Supplementary Fig. 1f, lanes 14 & 19). We concluded that the sA3G construct was eligible for structure analysis of A3G-Vif interaction.

Supplementary Note 2:

Discovery of RNA involvement in the A3G-Vif interaction

Previously, the Vif-binding determinants of A3G were identified by co-purification of sNTD variants and VCBC [1]; sNTD was expressed as a glutathione S-transferase (GST)-fused protein, and the sNTD-VCBC complex was harvested on a glutathione (GSH) resin. The assay was applied to four variants containing a mutated amino acid in the Vif-binding motif (Supplementary Fig. 2a and b). All mutants were co-purified with VCBC on GSH resin, except for sNTD-YFA (Supplementary Fig. 2b, lane 3), indicating the requirement of W127 for Vif-binding. When bound proteins were subjected to polyacrylamide gel electrophoresis and stained with a nucleotide-staining reagent, we found that all samples, except for sNTD-YFA, displayed a prominent band between the 100- and 200-bp markers

(Supplementary Fig. 2c). The band disappeared after treatment with RNase, whereas it was resistant to DNase (Supplementary Fig. 2d). These findings indicate that the harvested sNTD-VCBC complexes include an RNA component.

Next, we conducted RNA cloning and sequencing. The RNA component was extracted from the harvested sNTD-F126-VCBC complex and subjected to RNA cloning (see Methods). Sequences were obtained from seven clones (Supplementary Fig. 2e). All of them originated from the expression plasmid vector for Vif, and their sequences consistently corresponded to the 3′ parts of the respective open reading frame plus the following region. We focused on a consensus sequence in the open reading frame of Vif, nucleotide bases 604 to 657, and selected 30 nucleotides, designated RNA-I-30 (see Table I) which included a predicted stem-loop structure.

A synthetic RNA oligomer RNA-I-30 was prepared to test reconstitution of the sNTD-VCBC complex. As expected, the RNA-I-30 oligomer reproduced the sNTD-VCBC complex with individually purified proteins (Supplementary Fig. 2f). Even shorter RNA oligomers, such as RNA-I-20 and RNA-I-25, also mediated complex formation, although RNA-I-10 and RNA-I-15 apparently attenuated this capability (Supplementary Fig. 2f, lanes 4, 5 and Table I). We also tested complex formation using full-length A3G constructs with polynucleotide RNA-I-25 or DNA-T25 (Supplementary Fig. 2g and Table I). In the presence of DNA-T25, none of them produced the complex (Supplementary Fig. 2g, lanes 2-5). Three constructs, sNTD-F126-CTD, sNTD-AFW-CTD and sNTD-CTD, bind VCBC in the presence of RNA-I-25. In particular, sNTD-F126-CTD enhanced complex formation (Supplementary Fig. 2g, lanes 6-7), while no complex formation was observed for sNTD-YFA-CTD (Supplementary Fig. 2g, lane 8). These observations are consistent with the result of our co-expression assay using sNTD (Supplementary Fig. 2b). Taken together, we found RNA oligomers which successfully formed the A3G-VCBC complex.

Supplementary Note 3:

Structure-guided ligand RNA optimization by iterative cryo-EM analysis

We developed a solubility enhanced A3G variant, sA3G. In addition, we found an RNA fragment required to form a stable sNTD-VCBC complex in vitro. Our insights on A3G and RNA in combination with a Vif-CBFβ construct (VC) by Hu *et al.* [2] enabled us to prepare a stable complex, sA3G-VC-RNA (Supplementary Fig. 3a-c), and the purified complex resulted in a homogeneous particle distribution observed by negative staining TEM (Supplementary Fig. 3e).

First, we prepared the sA3G-VC complex using an RNA fragment RNA-I-20 (Supplementary Fig. 3a) and collected a cryo-EM dataset. Image processing steps followed a procedure shown in Supplementary Figure 4. The refined C2-symmetrized map indicated 4.2 Å resolution based on the Fourier shell correlation (FSC) using the 0.143 criterion (Supplementary Fig. 6b, c). The map clearly showed a β-sheet with a bump and a cylindrical feature at the center of complex

(Supplementary Fig. 6a, green), which fit a single-domain model of A3G nicely. It was assigned to the NTD, considering that the cylindrical map was followed by another A3G-shaped density, interpreted as sA3G CTD, which projects away from the center of the complex (Supplementary Fig. 6a, light green). Unexpectedly, the maps showed four VC components grouped around two sA3Gs, demonstrating that sA3G and VC form a complex with 1:2 stoichiometry in a C2 rotational symmetry (we designated the two VC components in a single asymmetrical unit as VC^{red} and VC^{blue}, as colored in Figure 1f). After fitting sA3G and VC models to the map, some map features still remained to be assigned (Supplementary Fig. 6a, yellow). These were most likely ligand RNA, although at this stage it remained uncertain.

We found further that another RNA oligomer, RNA-II-20, in which the 4th and 5th adenines were replaced by uracils, enhanced sA3G-VC complex formation (Supplementary Fig. 3b and Table I). The prepared complex sA3G-VC-RNA was subjected to cryo-EM analysis. Single-particle image data were processed with the previous map as an initial alignment reference (Supplementary Fig. 5a). The new cryo-EM map indicated improved resolution (Supplementary Fig. 6f, g). Although the map quality was not sufficient to discuss details of RNA binding to sA3G and VC, the RNA moiety appeared to pass through a narrow cavity between sA3G and VC^{blue}, and both of its ends contained relatively well-resolved features (Supplementary Fig. 6e and 9c, d). One end flanked by sA3G and VC^{red} and another end seemed to be accommodated in a shallow pocket of VC-blue (Supplementary Fig. 6e). Based on this interpretation, we presumed that the middle part of the RNA ligand may prefer pyrimidine bases to relieve steric hindrance and to fit into the narrow cavity between sA3G and VC^{blue}. As expected, complex formation was further increased when using an RNA oligomer, RNA-III-20, in which the 13th and 14th guanines were replaced with uracils (Supplementary Fig. 3c), whereas additional uracil replacement of the 9th and 10th nucleotides abolished this increase (Supplementary Fig. 3d, RNA-III-20a). Intriguingly, a drastic loss of complex formation was observed by removal of a single nucleotide at the center of the sequence (Supplementary Fig. 3d, RNA-III-20d). Truncations of peripheral nucleotides also decreased complex formation significantly (Supplementary Fig. 3d, RNA-III-20b and c). Thus, the ligand RNA prefers less bulkiness in its middle and requires sufficient length for efficient complex formation.

The RNA oligomer, RNA-III-20 (referred to as RNA20 hereafter and in the main text), was used for complex formation and cryo-EM data collection. Data processing followed a scheme described above (Supplementary Fig. 5b). The resulting 3D reconstruction reached 2.8 Å resolution and included the entire complex structure (Supplementary Fig. 6j, k). The map of the ligand RNA contained finer features and allowed conclusive assignment for some nucleotide bases, pyrimidine or purine, and the directionality of the polynucleotide. We concluded that the complex structure obtained using RNA20 had sufficient quality to warrant detailed interpretation of molecular features. Further results are discussed in the main text.

Supplementary Note 4:

Effect of rC-to-rU replacement on ligand RNA conformation

Additionally, we found that an RNA oligomer, RNA-IV-20, with a C9-to-U9 replacement even further enhances complex formation (Supplementary Fig. 3c and Table I). We collected a cryo-EM dataset and processed particle images using a similar scheme (Supplementary Fig. 5c). The resulting map indicated 2.5 Å resolution (Supplementary Fig. 6n, o). Although the protein arrangement was essentially identical to previous models (Supplementary Fig. 6m), the map suggests that replacement of C9-to-U9 caused a drastic change in the ligand RNA conformation (Supplementary Fig. 9i-l). The base, rC9, in RNA20 points toward the zinc-binding motif of sA3G (Supplementary Fig. 9i, j), whereas base rU9 in RNA-IV-20 is located away from the pocket and aligned parallel to base rC1 (Supplementary Fig. 9k, l). This enabled base rG10 to gain closer access to the surface of VC^{blue} and to establish a larger contact area, which seems to stabilize the VC^{blue}-RNA interaction. The atomic model of sA3G-VC-RNA-IV-20 also informed interpretation of unknown map features in the reconstruction of the sA3G-VC-RNA20 complex. After the atomic model of RNA20 was fit into the map, an extra density remained to be assigned (Supplementary Fig. 9i, asterisk). There was an obvious gap between the assigned and unassigned maps. (Supplementary Fig. 9i, arrow). Interestingly, the gap was not observed in the cryo-EM map of sA3G-VC-RNA-IV-20. Instead, another gap appeared at a location corresponding to a connection between rC9 and rG10 in the sA3G-VC-RNA20 model (Supplementary Fig. 9k, arrow). These alternating maps consistently support the hypothesis that C9-to-U9 replacement leads to a conformational change of the RNA. The map features corresponding to the unknown density in sA3G-VC-RNA20 fit confidently to nucleotide rG10 in sA3G-VC-RNA-IV-20. In addition, the unknown density in the sA3G-VC-RNA20 map can be assigned to rG10 of a minor RNA conformation. Thus, the map comparison between RNA20 and RNA-IV-20 suggests a local conformational change and a transient state around nucleotide rC9/rU9 of the ligand RNA. In contrast, other parts of the reconstruction, *i.e.,* nucleotides rC1 to rA7 and rU12 to rA18, were not significantly affected by the C9-to-U9 replacement (Supplementary Fig. 9m-p).

Supplementary Figure 1

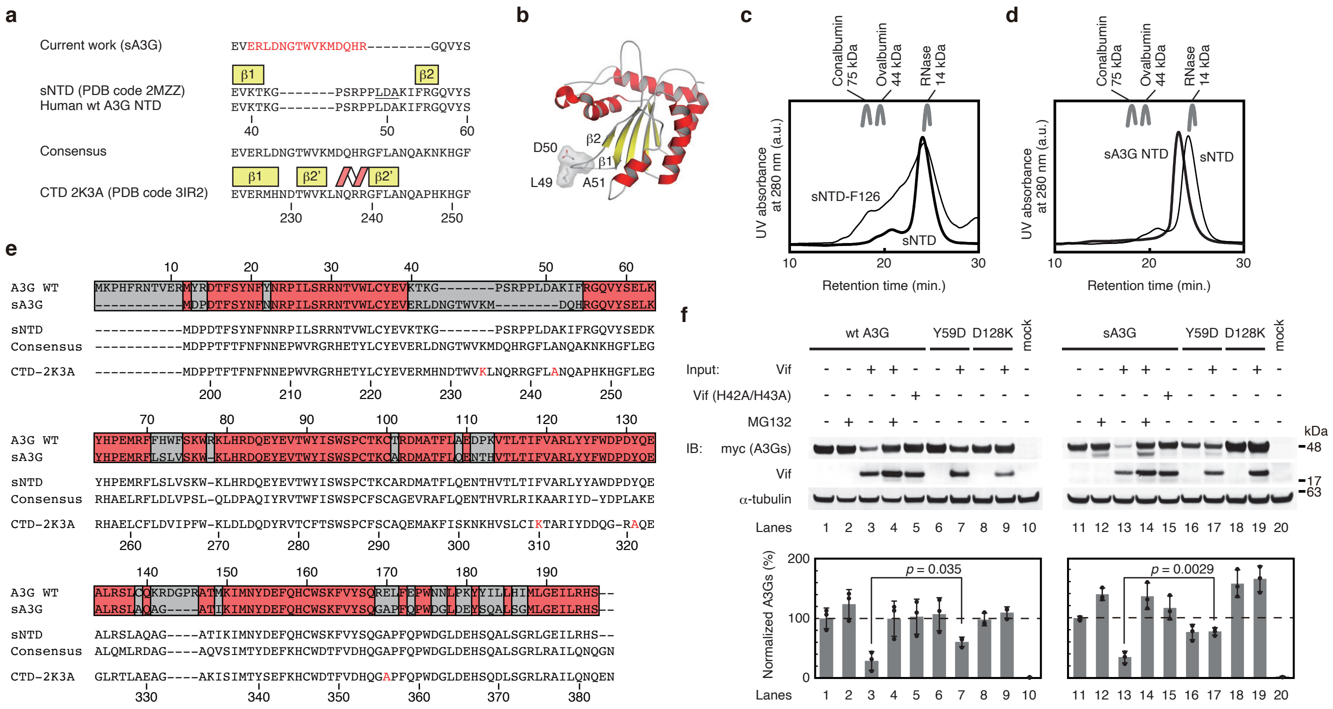

Supplementary Figure 1 | Preparation of solubility-enhanced A3G variant sA3G. **a**, Amino acid sequence alignment focused on the loop region β1/β2 of the sA3G NTD (current work), sNTD [23], wild-type A3G NTD, A3G CTD 2K3A [22], and a consensus construct [23]. Regions with α-helices and β-strands are marked with a red/white helical symbol and yellow boxes, respectively. Underlined amino acids, L49, D50 and A51 of sNTD, showed split NMR signals implying multiple conformations [23]. To stabilize the conformation, a consensus 15-amino acid sequence colored in red, ERLDNGTWVKMDQHR, was introduced into the sA3G construct. **b**, The NMR structure of sNTD is presented in ribbon representation [23]. Side chains of L49, D50 and A51 are drawn as stick models overlaid with their solvent-accessible surfaces. **c**, **d**, Size-exclusion chromatograms of sNTD (c, d), sNTD-F126 (c) and sA3G NTD (d). Peaks from molecular weight standards are shown above for reference. **e**, Amino acid sequence alignment of wild-type A3G NTD, sA3G NTD, sNTD, the consensus construct [23], and CTD-2K3A (5 mutations are indicated in red) [22]. The sA3G includes CTD-2K3A as CTD. Matching amino acids between wild-type A3G and sA3G are shaded in red, with mismatches in gray. Amino acid numbering follows the wild-type A3G NTD sequence and is used throughout the main text. **f**, Vif-induced A3G degradation assay using HEK293T cells. A series of wild-type (wt) A3G (left) and sA3G (right) constructs was tested with Vif or its H42A/H43A mutant. C-terminally myc-tagged A3G constructs, Vif and α-tubulin were specifically detected (upper panel). The density of detected A3G bands was quantified (lower panel). Both wild-type A3G and sA3G constructs gave essentially the same results. In the absence of Vif, both A3G constructs were expressed well, whereas their expression was decreased in its presence (lanes 1, 3, 11, and 13). The Vif-induced loss of A3G was abolished after adding the proteasome inhibitor, MG132 (lanes 2 and 12), or with Vif mutant H42A/H43A (lanes 5 and 15). Similarly, the mutant, A3G D128K, reduced A3G degradation in the presence of Vif (lanes 9 and 19). Substitution Y59D in A3G rescued A3G from the Vif-induced degradation significantly (lanes 7 and 17). Dashed center line of the box plot indicates normalized 100% A3G level. Dots represent individual data points. Assays were performed independently in triplicate (n=3). Error bars indicate standard deviation. Low p values ($p < 0.05$) from two-sided t-tests between the bracketed pairs (p=0.035 between lanes 3 and 7, p=0.0029 between lanes 13 and 17) attribute significance to their differences. Source data are provided with this paper.

Supplementary Figure 2

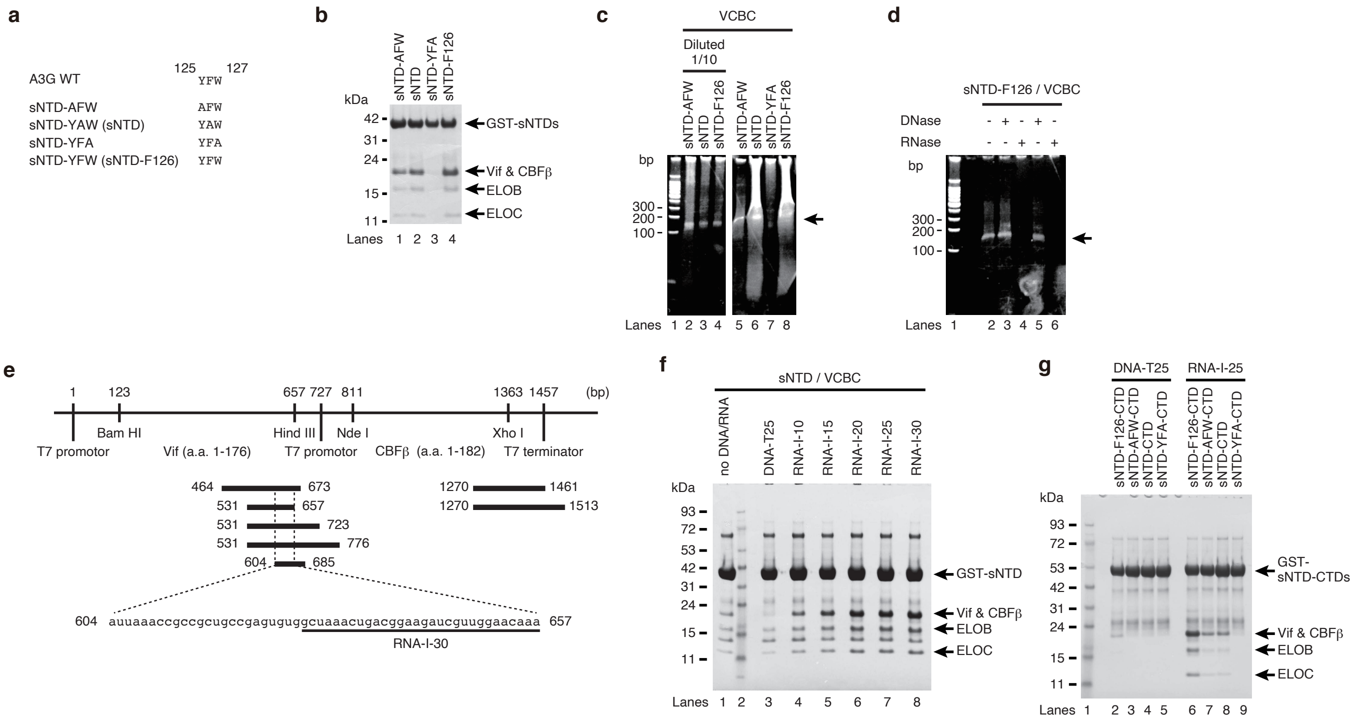

Supplementary Figure 2 | Discovery of RNA involvement in A3G-Vif interaction. **a**, Amino acid sequence comparison of a Vif-binding site (residues 125-127) from wild-type A3G and tested sNTD constructs. **b**, Pull-down assay using GST-fused sNTD constructs and VCBC. Harvested proteins were subjected to SDS-PAGE analysis. All sNTD constructs, except for sNTD-YFA (lane 3), co-purified with VCBC (lanes 1, 2, and 4). **c**, Protein-bound resins in panel (b) were also applied to PAGE and stained with the cyanine dye, SYBR green II. All sNTD constructs, except sNTD-YFA (lane 7), produced a band in the molecular range of 100-200 base pairs (lanes 2-6 and 8). **d**, DNase/RNase resistance of the unknown component obtained from the sNTD-F126-bound fraction (panel b). The obtained component was resistant to DNase treatment (lanes 3 and 5), whereas the band disappeared after RNase treatment (lanes 4 and 6). Representative gel image after a single time experiment is shown in (b-d). **e**, Mapping of seven sequences obtained from RNA cloning using the sNTD-F126-bound fraction (panel b). The vector structure is represented at the top. The lower, thick, horizontal lines represent obtained clone sequence regions, and the locations of their 5'- and 3'-edges are indicated by their base pair numbers. The consensus RNA sequence was obtained from five cloned sequences originating from the Vif open reading frame. Finally, a 30-base pair fragment, named RNA-I-30, was selected for further analyses. **f**, Pull-down assay of GST-fused sNTD and VCBC in the presence of synthesized polynucleotides. Harvested proteins were subjected to SDS-PAGE analysis. RNA-I-30 reproduced the sNTD-VCBC interaction (lane 8). As the polynucleotide was shortened, VCBC recovery was attenuated (lanes 4-7). In the absence of polynucleotide (lane 1), or in presence of DNA-T25 (lane 2), VCBC bands were barely detected. Representative gel image after a single time experiment is shown in (f-g). **g**, Pull-down assay of GST-fused full-length A3G variants and VCBC in the presence of synthesized polynucleotides. In the presence of DNA-T25, none of them produced VCBC bands (lanes 2-5), whereas in the presence of RNA-I-25, all of them, except sNTD-YFA-CTD (lane 9), produced VCBC bands (lanes 6-8). Source data are provided as a Source Data file.

Supplementary Figure 3

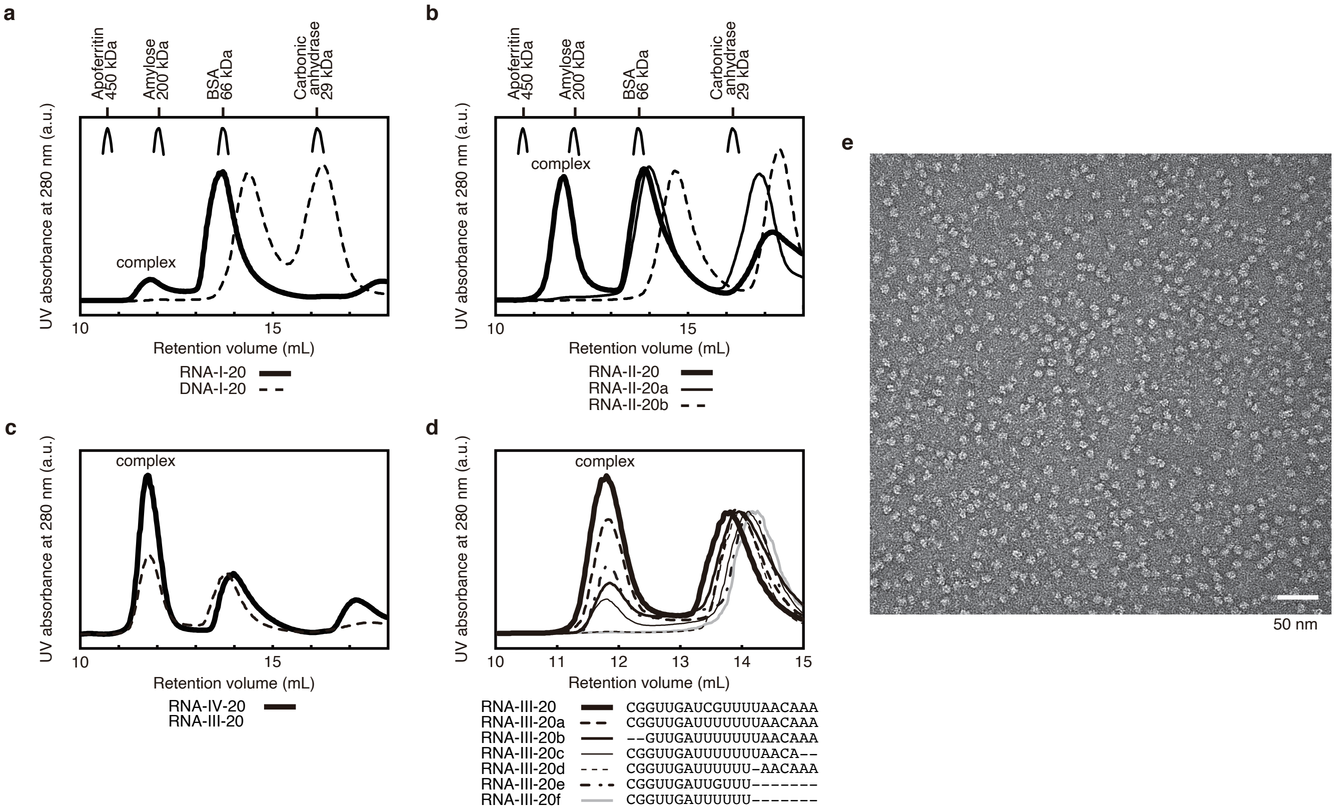

Supplementary Figure 3 | Preparation of the sA3G-VC-RNA complex and optimization of the RNA sequence. a-d, Size-exclusion chromatograms of a mixture of sA3G, VC, and synthesized DNA/RNA oligomers. Purified sA3G, VC, and DNA/RNA oligomer were always mixed at a ratio of 1:1.5:1.5, respectively. Each chromatogram was normalized against the peak height of free sA3G. Peaks of molecular weight standards are shown above. DNA/RNA oligomers employed are labeled beneath the chromatograms and their sequences are provided in Table I. RNA-I-20 produced a small peak for the sA3G-VC-RNA complex, whereas DNA-I-20 failed to form the complex (a). RNA-II-20 enhanced sA3G-VC-RNA complex formation, whereas neither RNA-II-20a nor RNA-II-20b formed the complex (b). RNA-III-20 (RNA20) and RNA-IV-20 further enhanced sA3G-VC-RNA complex formation (c). RNA-III-20a slightly decreased complex formation (d). RNA-III-20b and RNA-III-20c resulted in a substantial loss of complex formation ability. Removal of a single rU in the middle of RNA-III-20a almost completely abolished complex formation (RNA-III-20d). Interestingly, truncation of the 3'-side of the ligand loses complex formation ability (RNA-III-20f). The assembly can be partially rescued by introduction of rG in the middle (RNA-III-20e) (d). **e**, Representative transmission electron micrograph from 179 micrographs of the negatively stained complex, sA3G-VC-RNA20. Scale bar, 50 nm.

Supplementary Figure 4

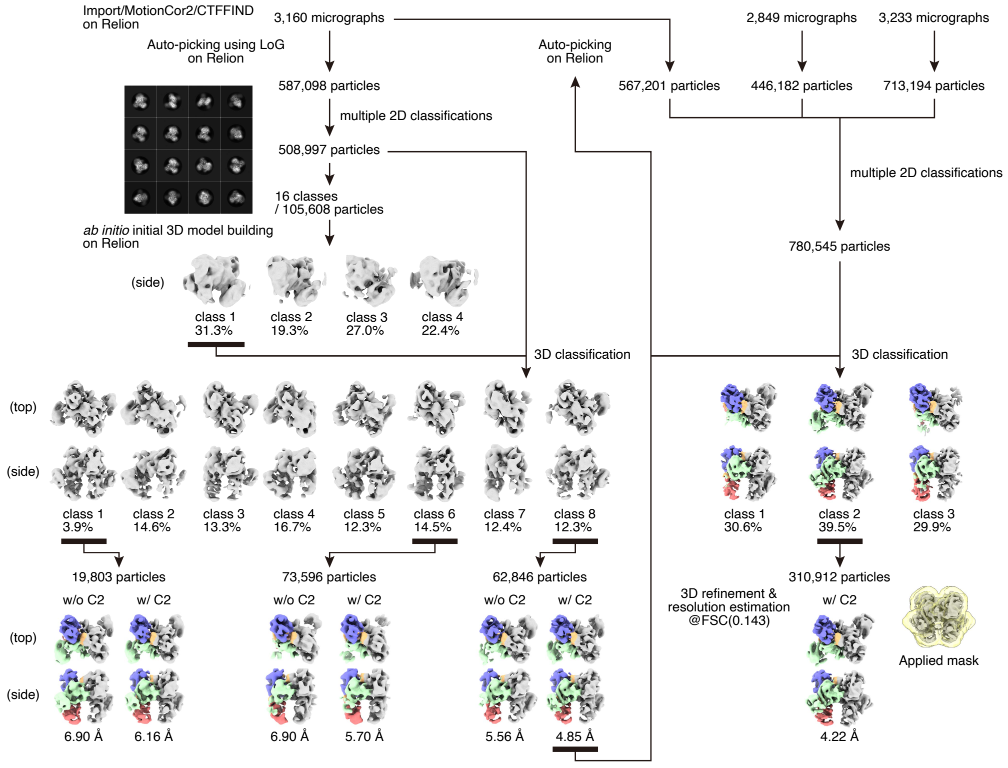

Supplementary Figure 4 | Cryo-EM image processing workflow using RELION for sA3G-VC-RNA-I-20 particle analysis. Movie data were collected with a Falcon 3EC direct electron detector in counting mode, on a Talos Arctica cryo-TEM operating at an acceleration voltage of 200 kV. Initially, 587,098 particles were obtained from 3,160 motion-corrected, dose-weighted micrographs. 105,608 selected particles were used for ab initio 3D reconstruction. After 3D classifications, 62,846 particles were selected for the initial 3D refinement. The refined reconstruction was used as an initial reference to process all data fractions. 1,726,577 particles were harvested from 9,242 micrographs. After 2D/3D classification, 310,912 particles were selected for 3D refinement with C2 symmetry, and the obtained map resolution was estimated at 4.2 Å, based on independent half-set refinement (Fourier Shell Correlation (FSC) = 0.143). The final map was used as a 3D reference for other single-particle analyses of sA3G-VC-RNA-II-20, sA3G-VC-RNA-III-20, and sA3G-VC-RNA-IV-20. All reference maps were lowpass-filtered in Fourier space to 1/20 Å.

Supplementary Figure 5

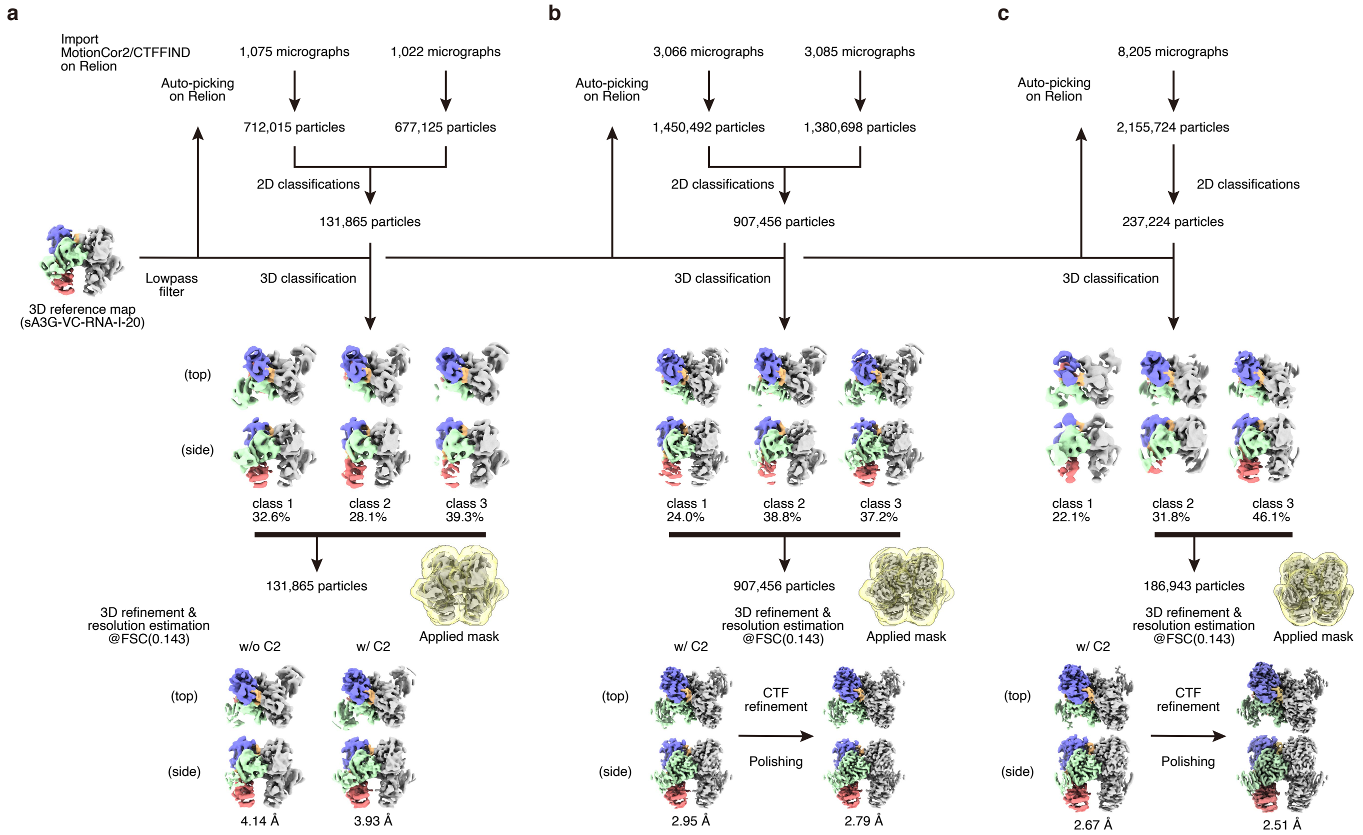

Supplementary Figure 5 | Cryo-EM image processing workflow using RELION for optimized sA3G-VC-RNA particles. Data processing schemes for single-particle analyses of sA3G-VC-RNA-II-20 complex (a), sA3G-VC-RNA-III-20 complex (b) and sA3G-VC-RNA-IV-20 complex (c). The final refined 3D map shown in Supplementary Figure 4 was used as an initial 3D reference for each analysis. **a**, Movie data were collected as described in Supplementary Figure 4. 1,389,140 particles were auto-picked from 2,097 micrographs with the lowpass-filtered 3D reference map. After 2D classification, 131,865 particles were selected. All selected particles were used for 3D refinement with C2-symmetry. Final map resolution was 3.9 Å. **b**, Movie data were collected on a Titan Krios cryo-TEM. 2,831,190 particles were auto-picked from 6,151 micrographs with the lowpass-filtered 3D reference map. After 2D classification, 907,456 particles were selected. At the end, all selected particles were used for final model refinement. The C2-symmetrized refined map resolution was estimated at 3.0 Å. Obtained particle images were subjected to iterative contrast transfer function (CTF) refinement and polishing. The final map resolution was 2.8 Å. This map was used to build an atomic model. **c**, Movie data were collected on a Titan Krios G4 TEM. 2,155,724 motion-corrected, dose-weighted particles were auto-picked from 8,205 micrographs with the lowpass-filtered 3D reference map. After 2D/3D classification, 187,050 particles were selected. Selected particles were used for a final model refinement. The resolution of the refined C2-symmetrized map was 2.7 Å. Obtained particle images were subjected to iterative CTF refinement and polishing. The final map resolution was 2.5 Å. Although Map (c) had higher nominal resolution than Map (b) and resolved the core of the structure slightly better, it did not change our interpretation of the RNA density. Because peripheral features were better represented in Map (b), the latter was used for Figure 1f. All reference maps were lowpass-filtered in Fourier space to 1/20 Å.

Supplementary Figure 6

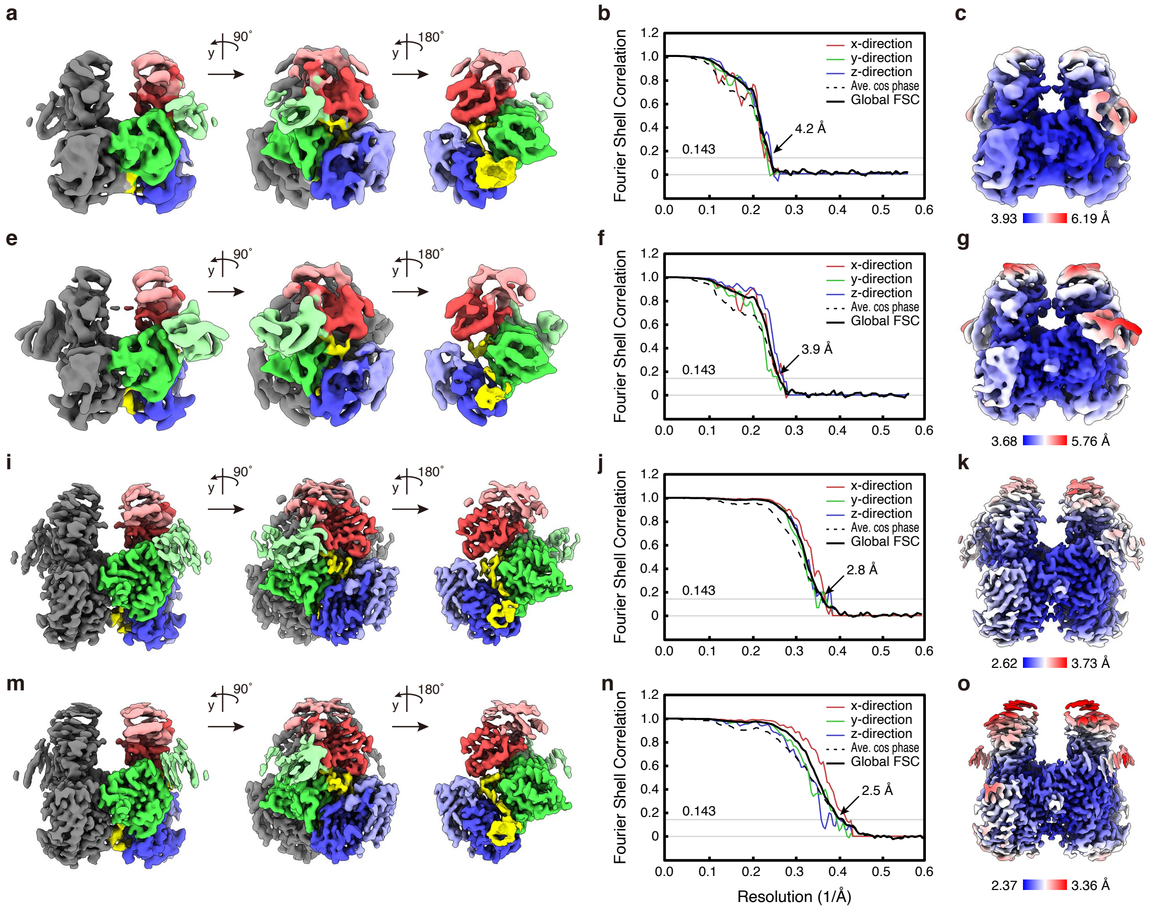

Supplementary Figure 6 | Obtained, refined maps and their evaluations. a-d, The complex of sA3G-VC-RNA-I-20. **e-h**, The complex of sA3G-VC-RNA-II-20. **i-l**, The complex of sA3G-VC-RNA-III-20 (RNA20). **m-p**, The complex of sA3G-VC-RNA-IV-20. Refined normalized maps were contoured at a threshold of 6σ (a, e, i, m). The rightmost map shows only one asymmetric unit. Coloring follows Figure 1f. Fourier shell correction curves calculated from independently refined unfiltered half-maps are shown (b, f, j, n). Calculated local resolution maps are drawn with a gradient from blue to red (c, g, k, o). The color range is provided beneath each map. Euler angle distribution of adapted particles (d, h, l, p).

Supplementary Figure 7

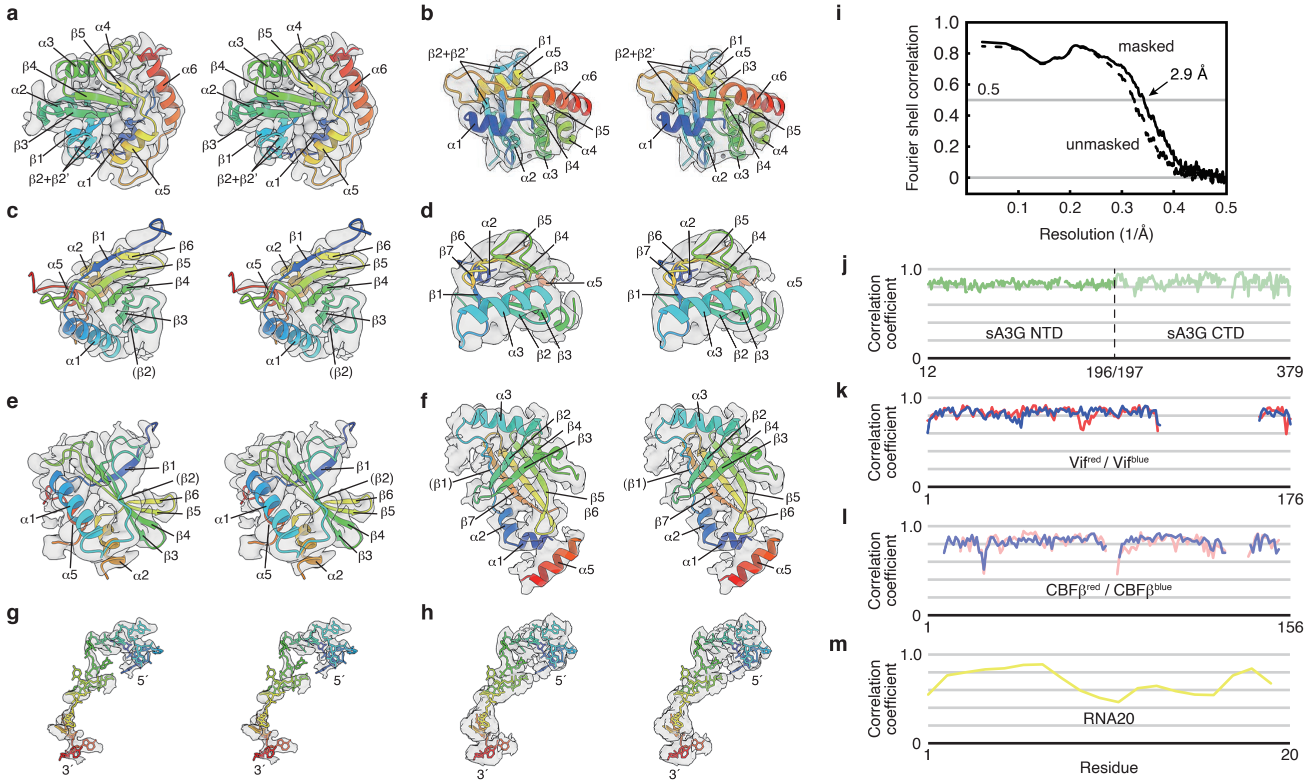

Supplementary Figure 7 | Evaluation of atomic model refinement and secondary structure elements of sA3G-VC-RNA20 complex. a-f, Stereo views of secondary structure elements superposed onto map isosurfaces of sA3G NTD (a) and CTD (b), Vif^{red} (c), $CBF\beta^{red}$ (d), Vif^{blue} (e) and $CBF\beta^{blue}$ (f). The molecular orientation follows Figure 1g. Polypeptides are rendered in ribbon representation with a color gradient from blue (N-terminus) to red (C-terminus). Normalized cryo-EM maps were contoured at a threshold of 6σ (a, c, e) or 4σ (b, d, f). **g, h**, Stereo views of maps and models of the RNA ligand RNA20. Polynucleotides are rendered in stick representation with a color gradient from blue (5´-end) to red (3´-end). Normalized cryo-EM maps were contoured at a threshold of 6σ (g) or 4σ (h). **i**, Fourier shell correlation curve calculated between the cryo-EM map and refined atomic model. Resolution at FSC = 0.5 was estimated at 2.9 Å. **j-m**, Correlation coefficient values per residue between map and model of sA3G (j), Vif^{red} and Vif^{blue} (k), $CBF\beta^{red}$ and $CBF\beta^{blue}$ (l) and RNA20 (m).

Supplementary Figure 8

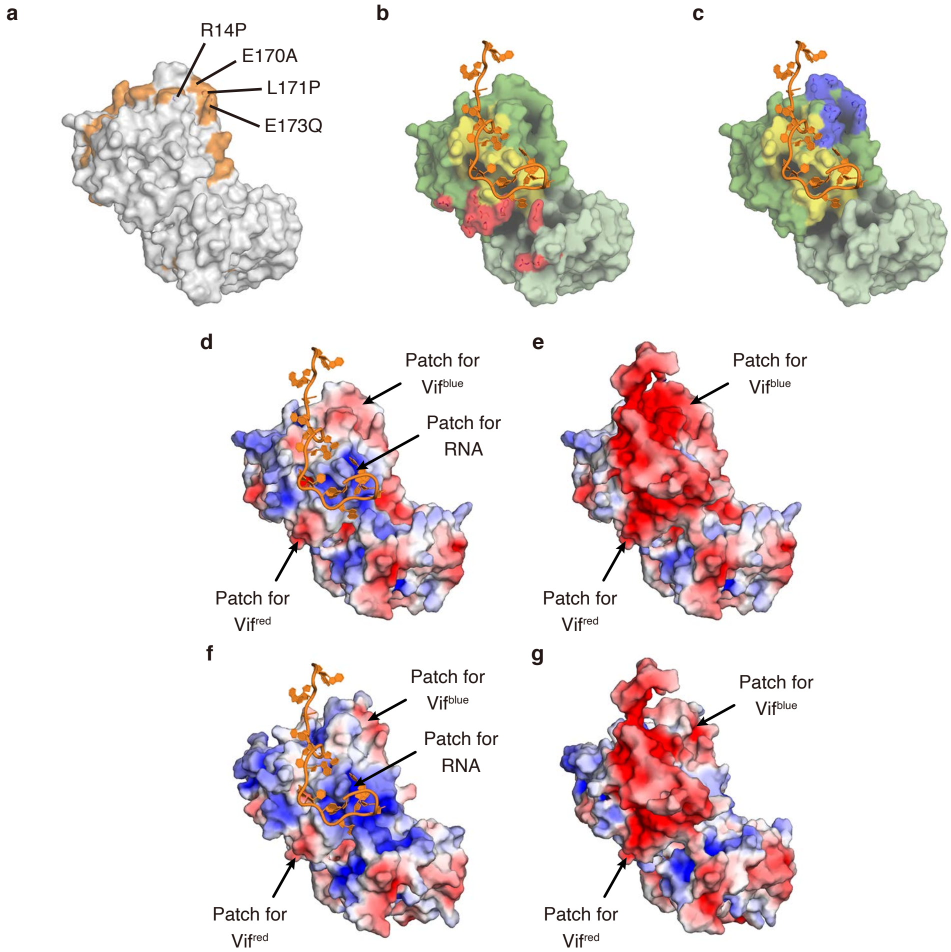

Supplementary Figure 8 | sA3G mutations and a predicted model of binding sites of wild type A3G. a, amino acid replacements introduced into sA3G variant. Solvent-accessible surface of sA3G is shown and the replaced amino acids are colored orange. Specifically, four replacements located in the sA3G-Vifblue interface are labeled. **b, c**, Binding interfaces identified in this study; sA3G-Vifred (b) and sA3G-Vifblue interfaces (c). Presentation follows figure 3b, d. **d-g**. Electrostatic potential surface distribution of sA3G (d, e) and wild type A3G (f, g). Panels d and f show sA3G and wild type A3G alone with the ligand RNA model while panels (e) and (g) represent A3G-RNA complex. Coloring follows figure 3f-i. Binding interface patches are labeled.

Supplementary Figure 9

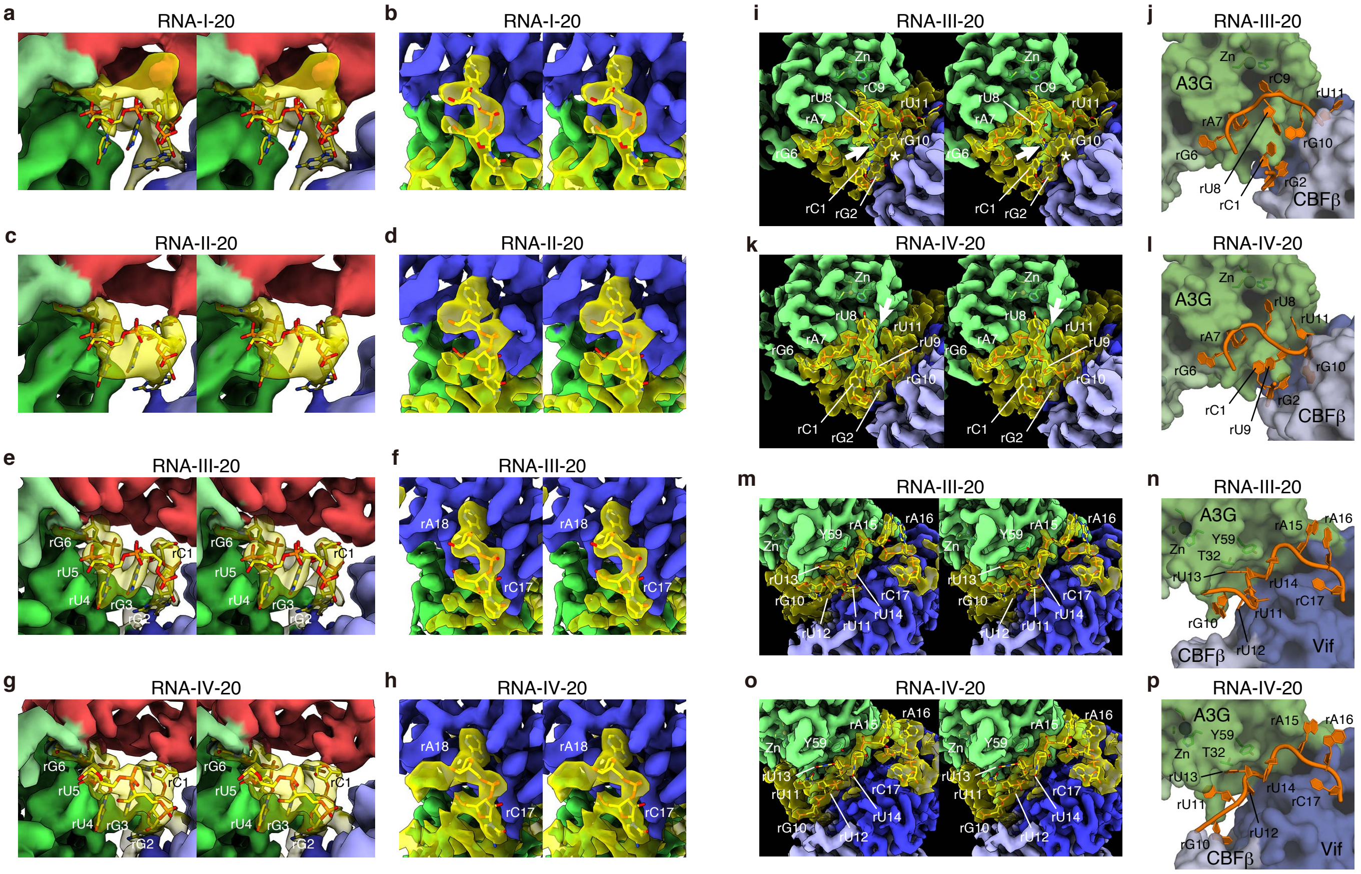

Supplementary Figure 9 | Close-up views of ligand RNA maps and models. Map coloring follows figure 1f. **a-h**, Stereo views of selected cryo-EM maps of sA3G-VC-RNA-I-20 (a, b), sA3G-VC-RNA-II-20 (c, d), sA3G-VC-RNA20 (e, f) and sA3G-VC-RNA-IV-20 (g, h) complexes. Refined maps were drawn at a threshold of 6σ to show nucleotides rC1 to rG6 (a, c, e, g), while sharpened maps were contoured at a threshold of 5σ (b, d) or 4σ (f, h) to show the dinucleotide, rC17rA18. The selected atomic model of sA3G-VC-RNA20 is represented as stick model and superimposed on the maps (a-f). The atomic model of sA3G-VC-RNA-IV-20 is shown as stick model and superimposed on the maps (g, h). rC17rA18 binding to Vifblue seems to be shared by all complexes, whereas the conformation of rC1 to rG6 appears to be variable. **i-p**, Close-up views of selected maps and models of sA3G-VC-RNA20 (i, j, m, n) and sA3G-VC-RNA-IV-20 (k, l, o, p) complexes. Nucleotides rC1, rG2, and rG6 to rU11 are focused in panels i-l, whereas nucleotides rG10 to rC17 are focused in panels m-p. The selected sharpened maps of proteins and ligand RNAs are shown at thresholds of 5σ and 3σ, respectively, in side-by-side stereo views (i, k, m, o). Corresponding proteins and RNAs are also drawn as a solvent-accessible surface and cartoon models, respectively (j, l, n, p). Discontinuities in map density are indicated by arrows (i, k) and an unassigned map feature is denoted by an asterisk (i). The location of sA3G amino acids T32 and Y59 are shown in panel (n, p). The zinc-binding motif is labeled as Zn. In summary, single-nucleotide replacement rC9-to-rU9 affected the conformations and relative positions of bases rC1, rU8, rC9/rU9, rG10 and rU11.

Supplementary Figure 10

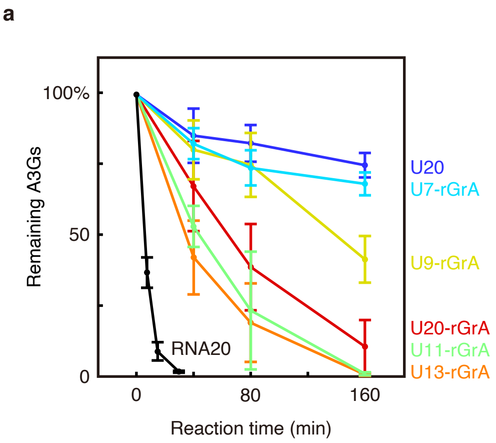

U20-rGrA	UUUUU GAUUU UUUUU UUUUU
U13-rGrA	UUUUU GAUUU UUU
U11-rGrA	UUUUU GAUUU U
U9-rGrA	UUUUU GAUU
U7-rGrA	UUU GAUU
U20	UUUUU UUUUU UUUUU UUUUU
RNA20	CGGUU GAUCG UUUUA ACAAA
Vif^{red} interaction	* ** *
Vif^{blue} interaction	* * ** * ***

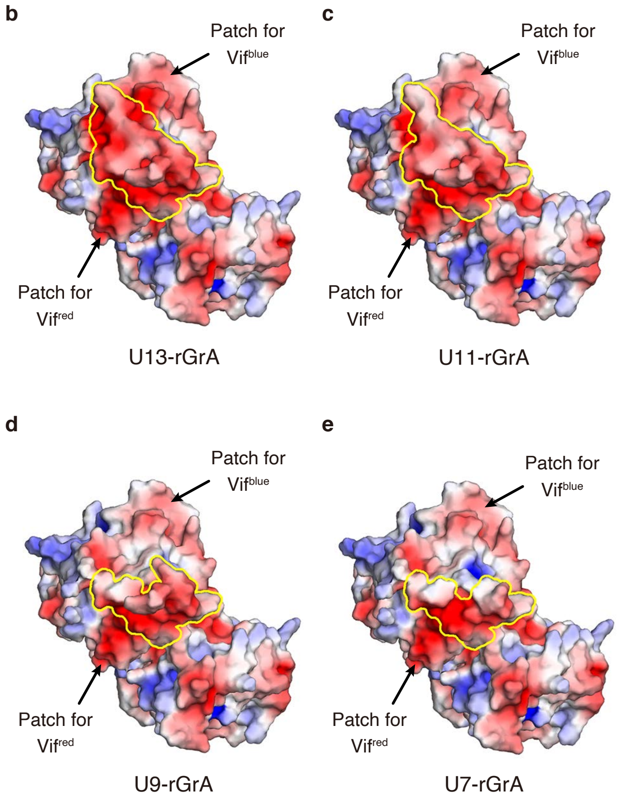

Supplementary Figure 10 | In-vitro ubiquitination assay using a series of 3´-side truncated RNA ligands. a, Effect on sA3G ubiquitination by shorter RNA ligands. U20-rGrA (sequence is shown below) enhanced sA3G ubiquitination. Likewise, U13-rGrA and U11-rGrA enhanced the reaction. While U9-rGrA showed a weak enhancement, U7-rGrA had no enhancement that was comparable to polyuridine nucleotide U20. Assays were performed independently in triplicate (n=3). Data points represent mean values. Error bars indicate standard deviation. Source data are provided with this paper. **b-e**, Predicted electrostatic potentials of sA3G in complex with U13-rGrA (b), U11-rGrA (c), U9-rGrA (d) and U7-rGrA (e). Red color indicates predominantly negative surface charge, white neutral, blue positive.

Supplementary Figure 11

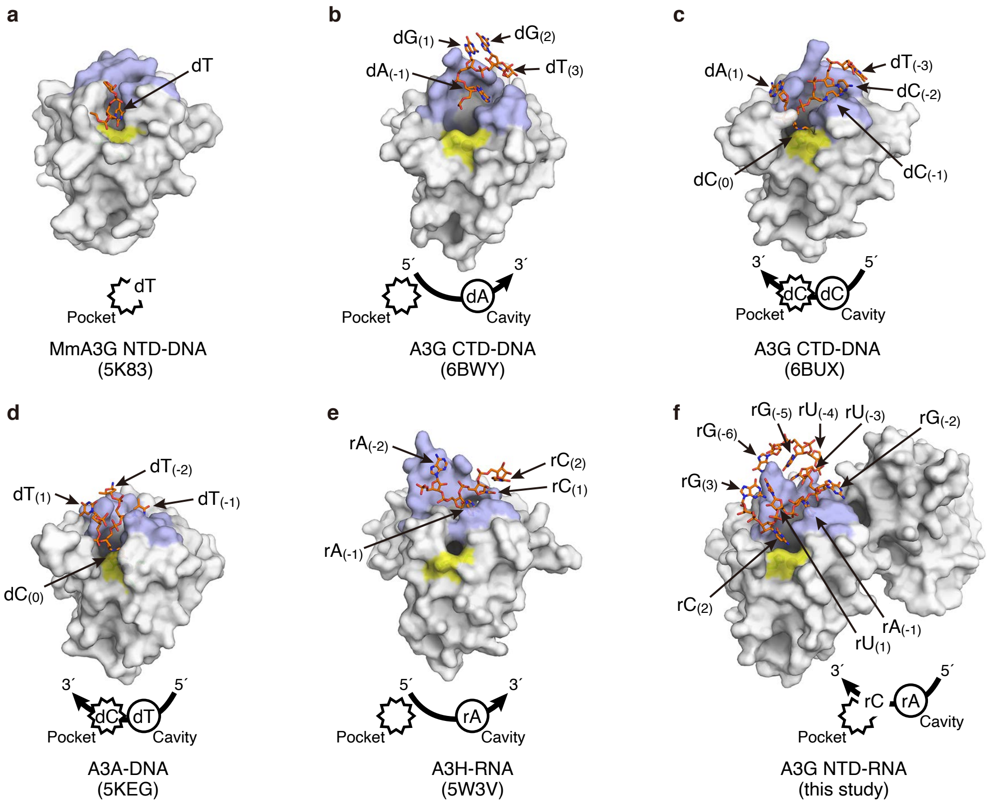

Supplementary Figure 11 | Structural comparison of the polynucleotide-binding mode of A3 proteins. Structure of primate A3G-DNA (PDB ID, 5K83; a), human A3G CTD-DNA (6BWY; b), human A3G CTD-DNA (6BUX; c), human A3A-DNA (5KEG; d), human A3H-RNA (5W3V; e) and sA3G-RNA complexes in this study (f). The solvent-accessible protein surface is presented with a ligand polynucleotide shown as a stick model. The surface colored in yellow represents a zinc-binding motif, whereas the surface cavity formed by $\alpha1/\beta1$ and $\beta4/\alpha4$ loops is in light purple. The nucleotide inserted into the active pocket is designated as position 0, and the preceding nucleotide accommodated in the surface cavity is assigned as position -1. If no nucleotide is in the pocket, position 0 was skipped. The nucleotide-binding mode of nucleotides at position -1 and 0 is demonstrated beneath the model.

Uncropped scans of all blots and gels in Figures are supplied in the Source Data file.

Supplementary References

1. Kouno, T., *et al.*, Structure of the Vif-binding domain of the antiviral enzyme APOBEC3G. *Nat. Struct. Mol. Biol.* **22**, 485-491 (2015).

2. Hu, Y., *et al.*, Structural basis of antagonism of human APOBEC3F by HIV-1 Vif. *Nat. Struct. Mol. Biol.* **26**, 1176-1183 (2019).

3. Chen, K.-M., *et al.*, Structure of the DNA deaminase domain of the HIV-1 restriction factor APOBEC3G. *Nature* **452**, 116-119 (2008).
