# Supplementary material for: Structural insights into RNA bridging between HIV-1 Vif and antiviral factor APOBEC3G
Source: Nat Commun. 2023 Jul 7;14:4037. doi: 10.1038/s41467-023-39796-5 (PMC10328928; doi:10.1038/s41467-023-39796-5)
Supplement: Supplementary file 3 — Reporting Summary [file 41467_2023_39796_MOESM3_ESM.pdf]

nature portfolio

Corresponding author(s): Matthias Wolf, Ph.D.
Takahide Kouno, Ph.D.

Last updated by author(s): Jun 18, 2023

Reporting Summary

Nature Portfolio wishes to improve the reproducibility of the work that we publish. This form provides structure for consistency and transparency in reporting. For further information on Nature Portfolio policies, see our Editorial Policies and the Editorial Policy Checklist.

Statistics

For all statistical analyses, confirm that the following items are present in the figure legend, table legend, main text, or Methods section.

n/a	Confirmed	
☐	☒	The exact sample size (*n*) for each experimental group/condition, given as a discrete number and unit of measurement
☐	☒	A statement on whether measurements were taken from distinct samples or whether the same sample was measured repeatedly
☐	☒	The statistical test(s) used AND whether they are one- or two-sided<br>*Only common tests should be described solely by name; describe more complex techniques in the Methods section.*
☒	☐	A description of all covariates tested
☒	☐	A description of any assumptions or corrections, such as tests of normality and adjustment for multiple comparisons
☐	☒	A full description of the statistical parameters including central tendency (e.g. means) or other basic estimates (e.g. regression coefficient) AND variation (e.g. standard deviation) or associated estimates of uncertainty (e.g. confidence intervals)
☐	☒	For null hypothesis testing, the test statistic (e.g. *F*, *t*, *r*) with confidence intervals, effect sizes, degrees of freedom and *P* value noted<br>*Give P values as exact values whenever suitable.*
☒	☐	For Bayesian analysis, information on the choice of priors and Markov chain Monte Carlo settings
☒	☐	For hierarchical and complex designs, identification of the appropriate level for tests and full reporting of outcomes
☒	☐	Estimates of effect sizes (e.g. Cohen's *d*, Pearson's *r*), indicating how they were calculated

Our web collection on statistics for biologists contains articles on many of the points above.

Software and code

Policy information about availability of computer code

Data collection	EPU 2.12.1
Data analysis	RELION 3.1.3<br>MotionCor2 1.3.0<br>Chimera 1.10.1<br>Chimera X 1.2.5<br>Phenix 1.19.2-4158<br>Pymol 2.3.4<br>Coot 0.8.9.2<br>MolProbity 4.5<br>Modeller 9.23<br>NAMD 2.13<br>VMD 1.9.4<br>NIH ImageJ 1.53i

For manuscripts utilizing custom algorithms or software that are central to the research but not yet described in published literature, software must be made available to editors and reviewers. We strongly encourage code deposition in a community repository (e.g. GitHub). See the Nature Portfolio guidelines for submitting code & software for further information.

Data

Policy information about availability of data

All manuscripts must include a data availability statement. This statement should provide the following information, where applicable:

- Accession codes, unique identifiers, or web links for publicly available datasets
- A description of any restrictions on data availability
- For clinical datasets or third party data, please ensure that the statement adheres to our policy

Atomic coordinates generated in this study for sA3G-VC-RNA20 and sA3G-VC-RNA-IV-20 were deposited in the Protein Data Bank under accession code 8H0I [https://doi.org/10.2210/pdb8h0i/pdb] (sA3G-VC-RNA20) and 8J62 [https://doi.org/10.2210/pdb8j62/pdb] (sA3G-VC-RNA-IV-20), respectively. Unsharpened cryo-EM density map, half-maps for sA3G-VC-RNA-I-20, sA3G-VC-RNA-II-20, sA3G-VC-RNA20 and sA3G-VC-RNA-IV-20 were deposited in the Electron Microscopy Data Bank under accession code EMD-35997 [https://www.ebi.ac.uk/emdb/EMD-35997] (sA3G-VC-RNA-I-20), EMD-35998 [https://www.ebi.ac.uk/emdb/EMD-35998] (sA3G-VC-RNA-II-20), EMD-34412 [https://www.ebi.ac.uk/emdb/EMD-34412] (sA3G-VC-RNA20) and EMD-35999 [https://www.ebi.ac.uk/emdb/EMD-35999] (sA3G-VC-RNA-IV-20), respectively (Table II). Source data are provided with this paper.

Human research participants

Policy information about studies involving human research participants and Sex and Gender in Research.

Reporting on sex and gender	N/A
Population characteristics	N/A
Recruitment	N/A
Ethics oversight	N/A

Note that full information on the approval of the study protocol must also be provided in the manuscript.

Field-specific reporting

Please select the one below that is the best fit for your research. If you are not sure, read the appropriate sections before making your selection.

☒ Life sciences ☐ Behavioural & social sciences ☐ Ecological, evolutionary & environmental sciences

For a reference copy of the document with all sections, see nature.com/documents/nr-reporting-summary-flat.pdf

Life sciences study design

All studies must disclose on these points even when the disclosure is negative.

Sample size	The sample size in cryo-EM is an individual single particle image. The total amount of single particle images was deemed adequate, since the inclusion of additional image data did not improve our cryo-EM reconstruction significantly. Biochemical experiments were carried out independently in triplicate, and all individual results are reported in the source data file.
Data exclusions	No data were excluded. All electron micrographs were analyzed. In cryo-EM image processing it is common practice to select subsets of the initial particle dataset to account for heterogeneity. The procedure was carried out unbiased and is described in detail in the methods.
Replication	As assessment of reproducibility, experimental replication was applied for in-vitro ubiquitination assay and Vif-induced A3G degradation assay (n=3). All attempts at replication were successful. Individual data points are reported in the source data file.
Randomization	There is no sample grouping in this study and therefore no randomization was applied.
Blinding	There is no sample grouping in this study and therefore no blinding was applied.

Reporting for specific materials, systems and methods

We require information from authors about some types of materials, experimental systems and methods used in many studies. Here, indicate whether each material, system or method listed is relevant to your study. If you are not sure if a list item applies to your research, read the appropriate section before selecting a response.

Materials & experimental systems

n/a	Involved in the study
☐	☒ Antibodies
☐	☒ Eukaryotic cell lines
☒	☐ Palaeontology and archaeology
☒	☐ Animals and other organisms
☒	☐ Clinical data
☒	☐ Dual use research of concern

Methods

n/a	Involved in the study
☒	☐ ChIP-seq
☒	☐ Flow cytometry
☒	☐ MRI-based neuroimaging

Antibodies

Antibodies used	monoclonal anti-c-myc tag antibody (Abcam #9E11), usage dilution 1:2000.<br>monoclonal anti-Vif antibody (Abcam #319), usage dilution 1:2000.<br>monoclonal anti-alpha-tubulin antibody (Abcam #DM1A), usage dilution 1:2000.<br>monoclonal anti-mouse IgG conjugated with a fluorophore IRDye 800CW (Abcam #ab216772), usage dilution 1:5000.
Validation	All primary antibodies were validated by the manufacture on their availability, at least, for western blotting application in terms of reactivity, specificity, and stability per lot. Also, we briefly confirmed that the antibodies detect purified antigen specifically, such as c-myc-fused A3G and Vif.

Eukaryotic cell lines

Policy information about cell lines and Sex and Gender in Research

Cell line source(s)	Human embryonic kidney (HEK) 293T cell line was used for protein expression. This cell line was originally obtained from Kawaoka Lab at Institute of Medical Science, University of Tokyo, and has been propagated in our lab.
Authentication	This cell line has previously been authenticated by DNA fIngerprinting (see https://www.nature.com/articles/s41586-018-0630-0)
Mycoplasma contamination	The cells tested negative for Mycoplasma contamination by Hoechst staining.
Commonly misidentified lines<br>(See ICLAC register)	No commonly misidentified cells were used in this study. The HEK293T strain is listed in the database. The cells were not the object of our study but used for protein expression only.
